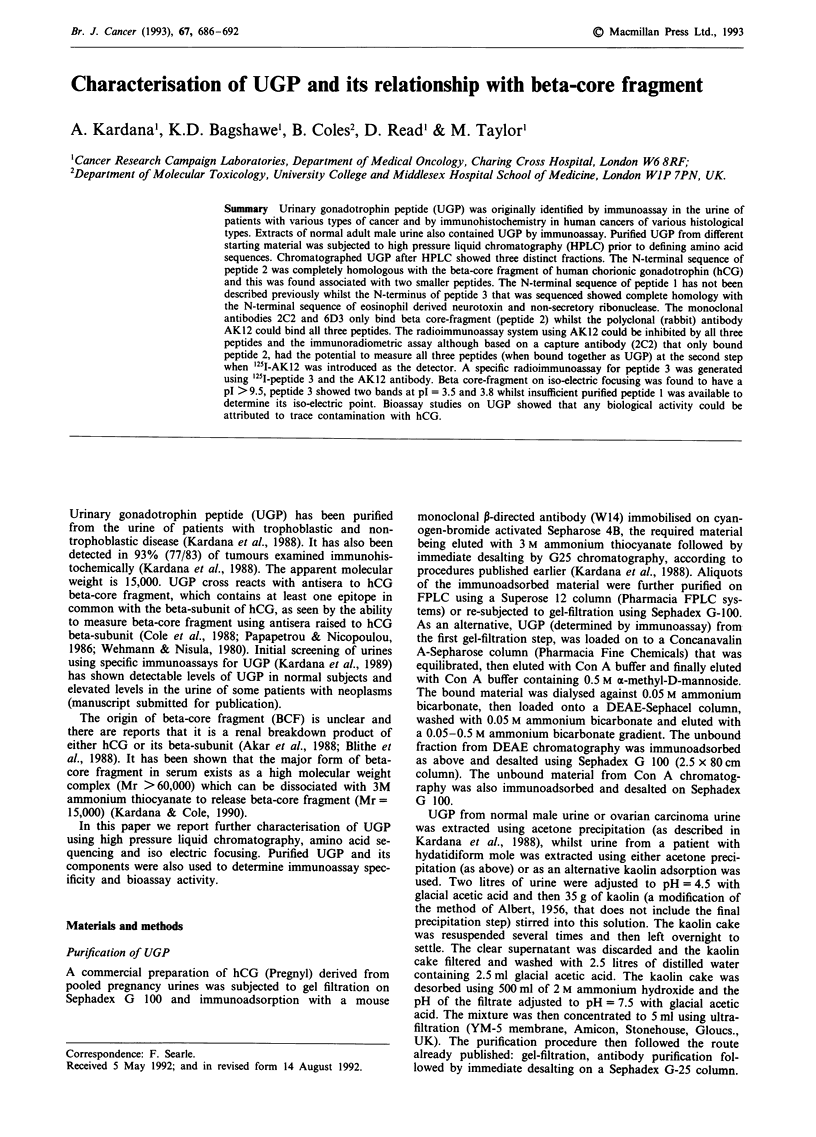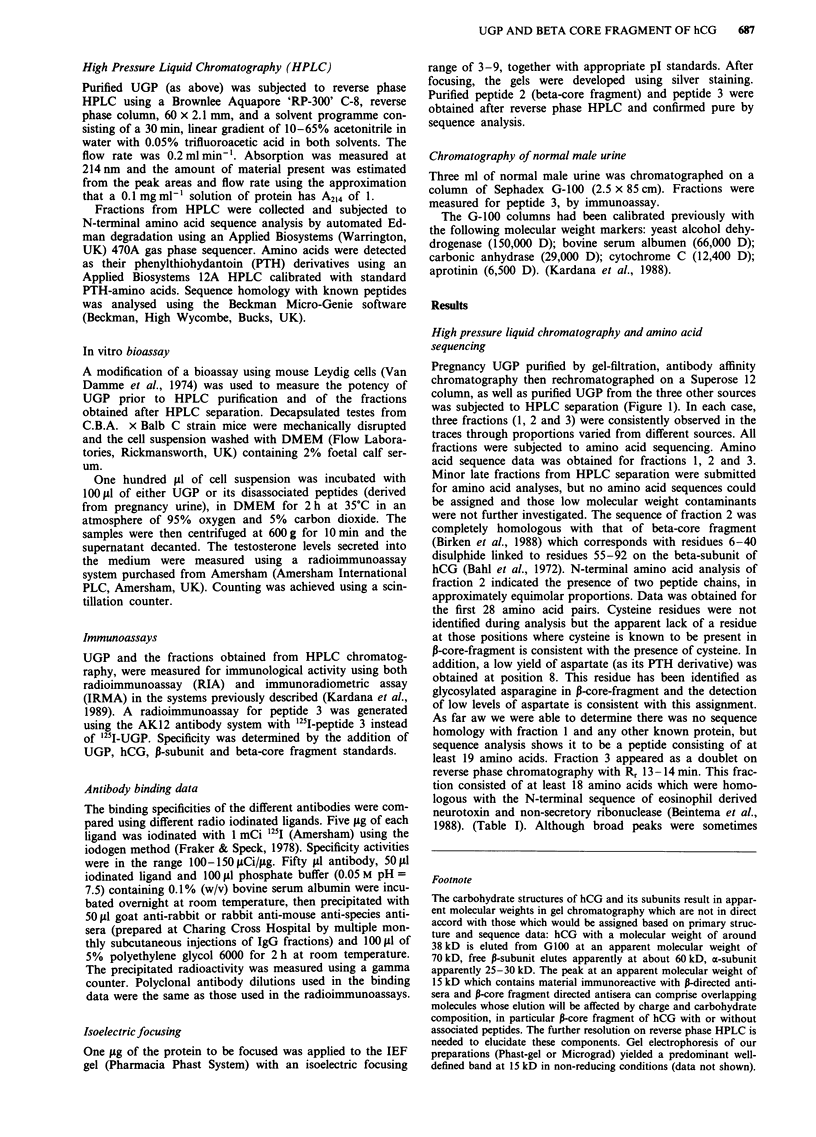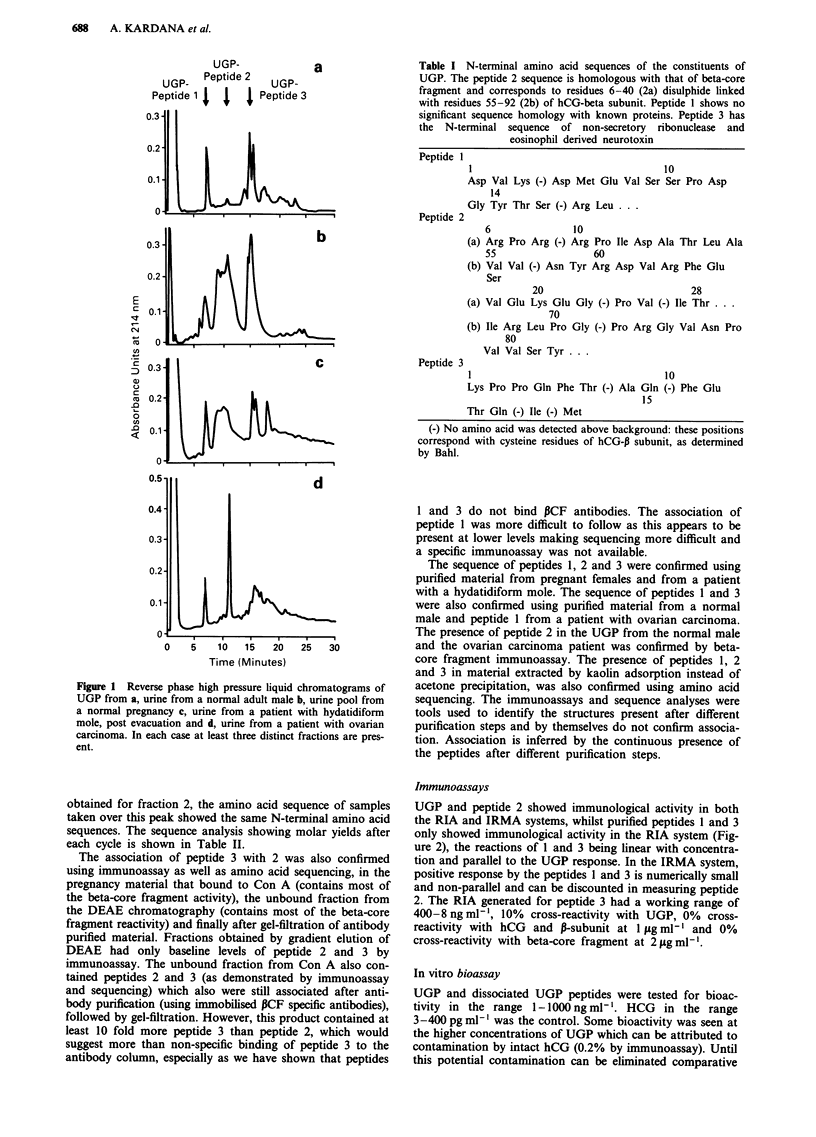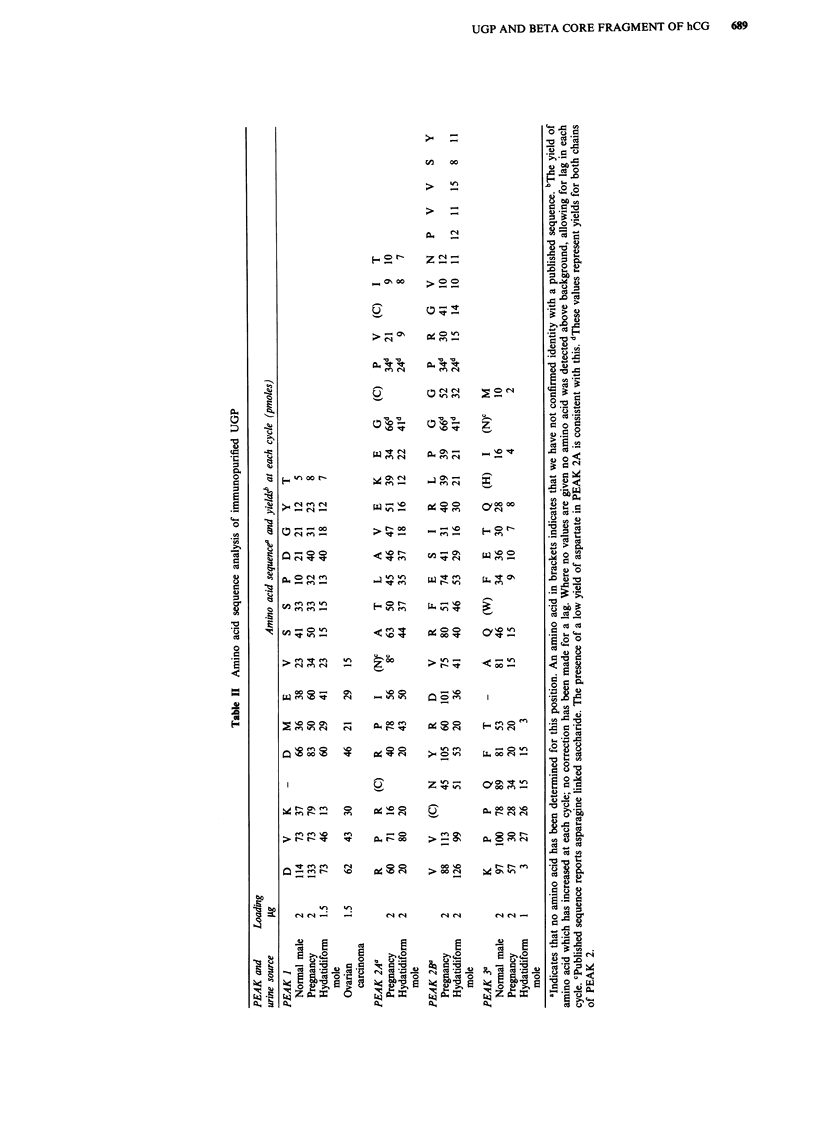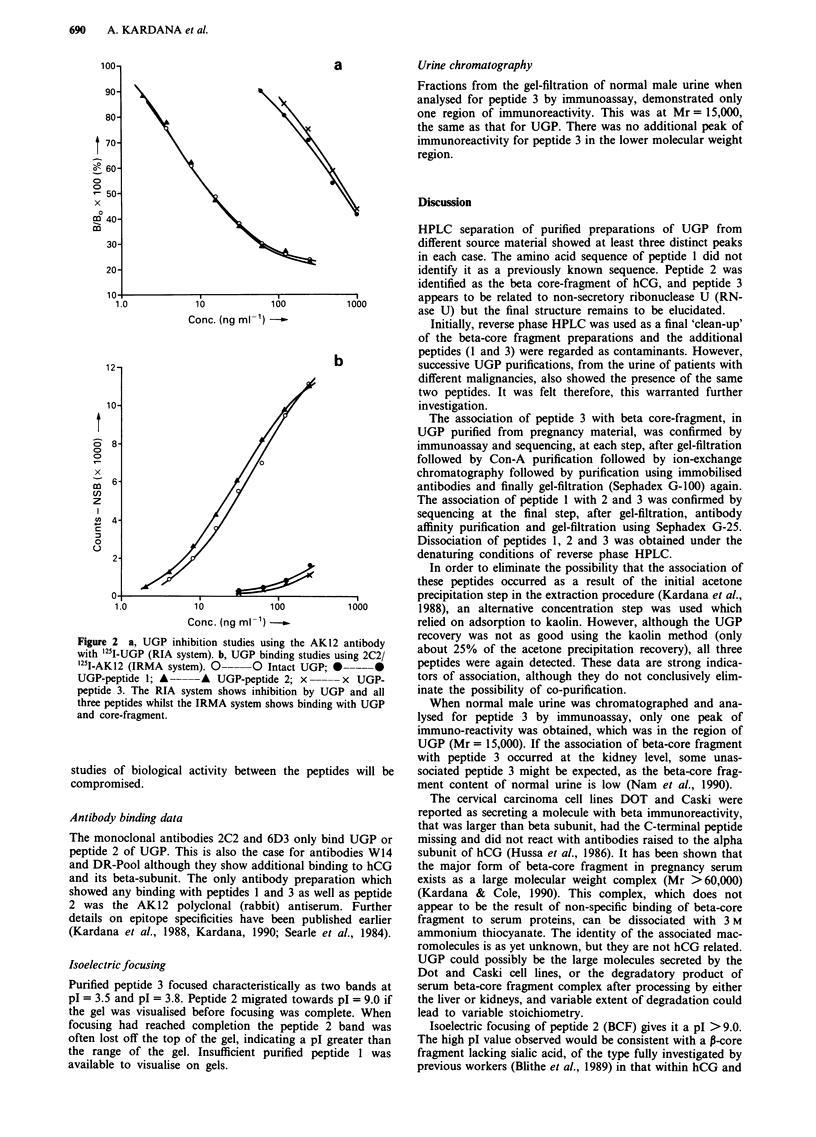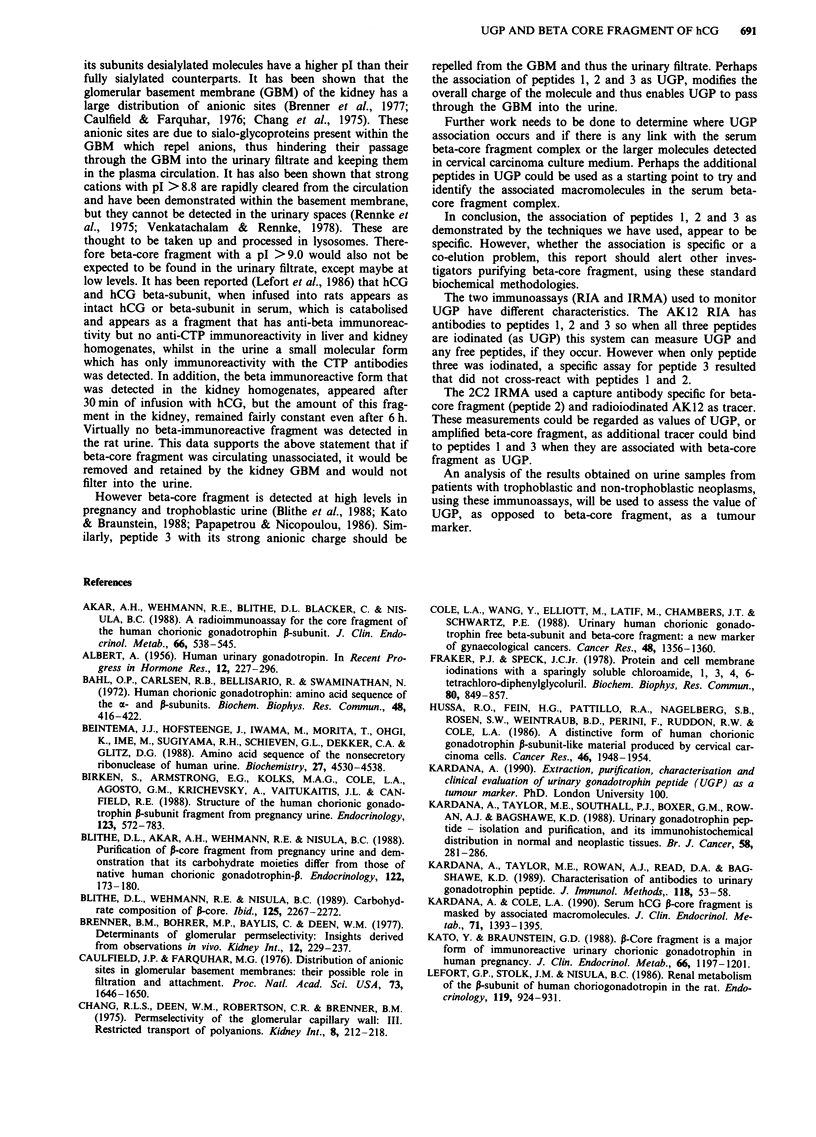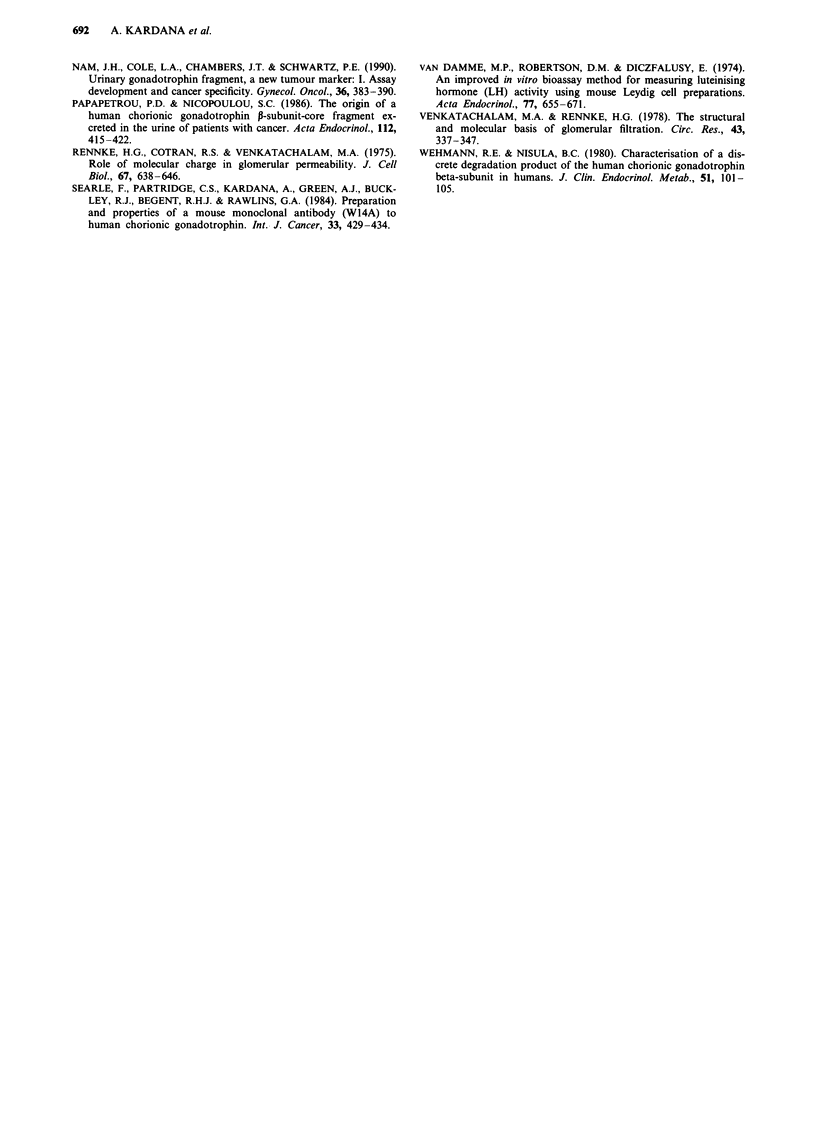# Characterisation of UGP and its relationship with beta-core fragment.

**DOI:** 10.1038/bjc.1993.127

**Published:** 1993-04

**Authors:** A. Kardana, K. D. Bagshawe, B. Coles, D. Read, M. Taylor

**Affiliations:** Department of Medical Oncology, Charing Cross Hospital, London, UK.

## Abstract

Urinary gonadotrophin peptide (UGP) was originally identified by immunoassay in the urine of patients with various types of cancer and by immunohistochemistry in human cancers of various histological types. Extracts of normal adult male urine also contained UGP by immunoassay. Purified UGP from different starting material was subjected to high pressure liquid chromatography (HPLC) prior to defining amino acid sequences. Chromatographed UGP after HPLC showed three distinct fractions. The N-terminal sequence of peptide 2 was completely homologous with the beta-core fragment of human chorionic gonadotrophin (hCG) and this was found associated with two smaller peptides. The N-terminal sequence of peptide 1 has not been described previously whilst the N-terminus of peptide 3 that was sequenced showed complete homology with the N-terminal sequence of eosinophil derived neurotoxin and non-secretory ribonuclease. The monoclonal antibodies 2C2 and 6D3 only bind beta core-fragment (peptide 2) whilst the polyclonal (rabbit) antibody AK12 could bind all three peptides. The radioimmunoassay system using AK12 could be inhibited by all three peptides and the immunoradiometric assay although based on a capture antibody (2C2) that only bound peptide 2, had the potential to measure all three peptides (when bound together as UGP) at the second step when 125I-AK12 was introduced as the detector. A specific radioimmunoassay for peptide 3 was generated using 125I-peptide 3 and the AK12 antibody. Beta core-fragment on iso-electric focusing was found to have a pI > 9.5, peptide 3 showed two bands at pI = 3.5 and 3.8 whilst insufficient purified peptide 1 was available to determine its iso-electric point. Bioassay studies on UGP showed that any biological activity could be attributed to trace contamination with hCG.


Br. J. Cancer (1993), 67, 686-692 © Macmillan Press Ltd., 1993

### Characterisation of UGP and its relationship with beta-core fragment

A. Kardana[1], K.D. Bagshawe[1], B. Coles[2], D. Read[1] & M. Taylor[1]

[1]*Cancer Research Campaign Laboratories, Department of Medical Oncology, Charing Cross Hospital, London W6 8RF;*
[2]*Department of Molecular Toxicology, University College and Middlesex Hospital School of Medicine, London W1P 7PN, UK.*

**Summary** Urinary gonadotrophin peptide (UGP) was originally identified by immunoassay in the urine of patients with various types of cancer and by immunohistochemistry in human cancers of various histological types. Extracts of normal adult male urine also contained UGP by immunoassay. Purified UGP from different starting material was subjected to high pressure liquid chromatography (HPLC) prior to defining amino acid sequences. Chromatographed UGP after HPLC showed three distinct fractions. The N-terminal sequence of peptide 2 was completely homologous with the beta-core fragment of human chorionic gonadotrophin (hCG) and this was found associated with two smaller peptides. The N-terminal sequence of peptide 1 has not been described previously whilst the N-terminus of peptide 3 that was sequenced showed complete homology with the N-terminal sequence of eosinophil derived neurotoxin and non-secretory ribonuclease. The monoclonal antibodies 2C2 and 6D3 only bind beta core-fragment (peptide 2) whilst the polyclonal (rabbit) antibody AK12 could bind all three peptides. The radioimmunoassay system using AK12 could be inhibited by all three peptides and the immunoradiometric assay although based on a capture antibody (2C2) that only bound peptide 2, had the potential to measure all three peptides (when bound together as UGP) at the second step when $^{125}$I-AK12 was introduced as the detector. A specific radioimmunoassay for peptide 3 was generated using $^{125}$I-peptide 3 and the AK12 antibody. Beta core-fragment on iso-electric focusing was found to have a pI $>9.5$, peptide 3 showed two bands at pI = 3.5 and 3.8 whilst insufficient purified peptide 1 was available to determine its iso-electric point. Bioassay studies on UGP showed that any biological activity could be attributed to trace contamination with hCG.

Urinary gonadotrophin peptide (UGP) has been purified from the urine of patients with trophoblastic and non-trophoblastic disease (Kardana *et al.*, 1988). It has also been detected in 93% (77/83) of tumours examined immunohistochemically (Kardana *et al.*, 1988). The apparent molecular weight is 15,000. UGP cross reacts with antisera to hCG beta-core fragment, which contains at least one epitope in common with the beta-subunit of hCG, as seen by the ability to measure beta-core fragment using antisera raised to hCG beta-subunit (Cole *et al.*, 1988; Papapetrou & Nicopoulou, 1986; Wehmann & Nisula, 1980). Initial screening of urines using specific immunoassays for UGP (Kardana *et al.*, 1989) has shown detectable levels of UGP in normal subjects and elevated levels in the urine of some patients with neoplasms (manuscript submitted for publication).

The origin of beta-core fragment (BCF) is unclear and there are reports that it is a renal breakdown product of either hCG or its beta-subunit (Akar *et al.*, 1988; Blithe *et al.*, 1988). It has been shown that the major form of beta-core fragment in serum exists as a high molecular weight complex (Mr $>60,000$) which can be dissociated with 3M ammonium thiocyanate to release beta-core fragment (Mr = 15,000) (Kardana & Cole, 1990).

In this paper we report further characterisation of UGP using high pressure liquid chromatography, amino acid sequencing and iso electric focusing. Purified UGP and its components were also used to determine immunoassay specificity and bioassay activity.

### Materials and methods

### *Purification of UGP*

A commercial preparation of hCG (Pregnyl) derived from pooled pregnancy urines was subjected to gel filtration on Sephadex G 100 and immunoadsorption with a mouse monoclonal β-directed antibody (W14) immobilised on cyanogen-bromide activated Sepharose 4B, the required material being eluted with 3 M ammonium thiocyanate followed by immediate desalting by G25 chromatography, according to procedures published earlier (Kardana *et al.*, 1988). Aliquots of the immunoadsorbed material were further purified on FPLC using a Superose 12 column (Pharmacia FPLC systems) or re-subjected to gel-filtration using Sephadex G-100. As an alternative, UGP (determined by immunoassay) from the first gel-filtration step, was loaded on to a Concanavalin A-Sepharose column (Pharmacia Fine Chemicals) that was equilibrated, then eluted with Con A buffer and finally eluted with Con A buffer containing 0.5 M α-methyl-D-mannoside. The bound material was dialysed against 0.05 M ammonium bicarbonate, then loaded onto a DEAE-Sephacel column, washed with 0.05 M ammonium bicarbonate and eluted with a 0.05–0.5 M ammonium bicarbonate gradient. The unbound fraction from DEAE chromatography was immunoadsorbed as above and desalted using Sephadex G 100 (2.5 × 80 cm column). The unbound material from Con A chromatography was also immunoadsorbed and desalted on Sephadex G 100.

UGP from normal male urine or ovarian carcinoma urine was extracted using acetone precipitation (as described in Kardana *et al.*, 1988), whilst urine from a patient with hydatidiform mole was extracted using either acetone precipitation (as above) or as an alternative kaolin adsorption was used. Two litres of urine were adjusted to pH = 4.5 with glacial acetic acid and then 35 g of kaolin (a modification of the method of Albert, 1956, that does not include the final precipitation step) stirred into this solution. The kaolin cake was resuspended several times and then left overnight to settle. The clear supernatant was discarded and the kaolin cake filtered and washed with 2.5 litres of distilled water containing 2.5 ml glacial acetic acid. The kaolin cake was desorbed using 500 ml of 2 M ammonium hydroxide and the pH of the filtrate adjusted to pH = 7.5 with glacial acetic acid. The mixture was then concentrated to 5 ml using ultrafiltration (YM-5 membrane, Amicon, Stonehouse, Gloucs., UK). The purification procedure then followed the route already published: gel-filtration, antibody purification followed by immediate desalting on a Sephadex G-25 column.

Correspondence: F. Searle.
Received 5 May 1992; and in revised form 14 August 1992.

*High Pressure Liquid Chromatography (HPLC)*

Purified UGP (as above) was subjected to reverse phase HPLC using a Brownlee Aquapore 'RP-300' C-8, reverse phase column, 60 × 2.1 mm, and a solvent programme consisting of a 30 min, linear gradient of 10–65% acetonitrile in water with 0.05% trifluoroacetic acid in both solvents. The flow rate was 0.2 ml $min^{-1}$. Absorption was measured at 214 nm and the amount of material present was estimated from the peak areas and flow rate using the approximation that a 0.1 mg $ml^{-1}$ solution of protein has $A_{214}$ of 1.

Fractions from HPLC were collected and subjected to N-terminal amino acid sequence analysis by automated Edman degradation using an Applied Biosystems (Warrington, UK) 470A gas phase sequencer. Amino acids were detected as their phenylthiohydantoin (PTH) derivatives using an Applied Biosystems 12A HPLC calibrated with standard PTH-amino acids. Sequence homology with known peptides was analysed using the Beckman Micro-Genie software (Beckman, High Wycombe, Bucks, UK).

In vitro *bioassay*

A modification of a bioassay using mouse Leydig cells (Van Damme *et al.*, 1974) was used to measure the potency of UGP prior to HPLC purification and of the fractions obtained after HPLC separation. Decapsulated testes from C.B.A. × Balb C strain mice were mechanically disrupted and the cell suspension washed with DMEM (Flow Laboratories, Rickmansworth, UK) containing 2% foetal calf serum.

One hundred µl of cell suspension was incubated with 100 µl of either UGP or its disassociated peptides (derived from pregnancy urine), in DMEM for 2 h at 35°C in an atmosphere of 95% oxygen and 5% carbon dioxide. The samples were then centrifuged at 600 g for 10 min and the supernatant decanted. The testosterone levels secreted into the medium were measured using a radioimmunoassay system purchased from Amersham (Amersham International PLC, Amersham, UK). Counting was achieved using a scintillation counter.

*Immunoassays*

UGP and the fractions obtained from HPLC chromatography, were measured for immunological activity using both radioimmunoassay (RIA) and immunoradiometric assay (IRMA) in the systems previously described (Kardana *et al.*, 1989). A radioimmunoassay for peptide 3 was generated using the AK12 antibody system with $^{125}$I-peptide 3 instead of $^{125}$I-UGP. Specificity was determined by the addition of UGP, hCG, β-subunit and beta-core fragment standards.

*Antibody binding data*

The binding specificities of the different antibodies were compared using different radio iodinated ligands. Five µg of each ligand was iodinated with 1 mCi $^{125}$I (Amersham) using the iodogen method (Fraker & Speck, 1978). Specificity activities were in the range 100–150 µCi/µg. Fifty µl antibody, 50 µl iodinated ligand and 100 µl phosphate buffer (0.05 M pH = 7.5) containing 0.1% (w/v) bovine serum albumin were incubated overnight at room temperature, then precipitated with 50 µl goat anti-rabbit or rabbit anti-mouse anti-species antisera (prepared at Charing Cross Hospital by multiple monthly subcutaneous injections of IgG fractions) and 100 µl of 5% polyethylene glycol 6000 for 2 h at room temperature. The precipitated radioactivity was measured using a gamma counter. Polyclonal antibody dilutions used in the binding data were the same as those used in the radioimmunoassays.

*Isoelectric focusing*

One µg of the protein to be focused was applied to the IEF gel (Pharmacia Phast System) with an isoelectric focusing range of 3–9, together with appropriate pI standards. After focusing, the gels were developed using silver staining. Purified peptide 2 (beta-core fragment) and peptide 3 were obtained after reverse phase HPLC and confirmed pure by sequence analysis.

*Chromatography of normal male urine*

Three ml of normal male urine was chromatographed on a column of Sephadex G-100 (2.5 × 85 cm). Fractions were measured for peptide 3, by immunoassay.

The G-100 columns had been calibrated previously with the following molecular weight markers: yeast alcohol dehydrogenase (150,000 D); bovine serum albumen (66,000 D); carbonic anhydrase (29,000 D); cytochrome C (12,400 D); aprotinin (6,500 D). (Kardana *et al.*, 1988).

### Results

*High pressure liquid chromatography and amino acid sequencing*

Pregnancy UGP purified by gel-filtration, antibody affinity chromatography then rechromatographed on a Superose 12 column, as well as purified UGP from the three other sources was subjected to HPLC separation (Figure 1). In each case, three fractions (1, 2 and 3) were consistently observed in the traces through proportions varied from different sources. All fractions were subjected to amino acid sequencing. Amino acid sequence data was obtained for fractions 1, 2 and 3. Minor late fractions from HPLC separation were submitted for amino acid analyses, but no amino acid sequences could be assigned and those low molecular weight contaminants were not further investigated. The sequence of fraction 2 was completely homologous with that of beta-core fragment (Birken *et al.*, 1988) which corresponds with residues 6–40 disulphide linked to residues 55–92 on the beta-subunit of hCG (Bahl *et al.*, 1972). N-terminal amino acid analysis of fraction 2 indicated the presence of two peptide chains, in approximately equimolar proportions. Data was obtained for the first 28 amino acid pairs. Cysteine residues were not identified during analysis but the apparent lack of a residue at those positions where cysteine is known to be present in β-core-fragment is consistent with the presence of cysteine. In addition, a low yield of aspartate (as its PTH derivative) was obtained at position 8. This residue has been identified as glycosylated asparagine in β-core-fragment and the detection of low levels of aspartate is consistent with this assignment. As far aw we were able to determine there was no sequence homology with fraction 1 and any other known protein, but sequence analysis shows it to be a peptide consisting of at least 19 amino acids. Fraction 3 appeared as a doublet on reverse phase chromatography with $R_r$ 13–14 min. This fraction consisted of at least 18 amino acids which were homologous with the N-terminal sequence of eosinophil derived neurotoxin and non-secretory ribonuclease (Beintema *et al.*, 1988). (Table I). Although broad peaks were sometimes

*Footnote*

The carbohydrate structures of hCG and its subunits result in apparent molecular weights in gel chromatography which are not in direct accord with those which would be assigned based on primary structure and sequence data: hCG with a molecular weight of around 38 kD is eluted from G100 at an apparent molecular weight of 70 kD, free β-subunit elutes apparently at about 60 kD, α-subunit apparently 25–30 kD. The peak at an apparent molecular weight of 15 kD which contains material immunoreactive with β-directed antisera and β-core fragment directed antisera can comprise overlapping molecules whose elution will be affected by charge and carbohydrate composition, in particular β-core fragment of hCG with or without associated peptides. The further resolution on reverse phase HPLC is needed to elucidate these components. Gel electrophoresis of our preparations (Phast-gel or Micrograd) yielded a predominant well-defined band at 15 kD in non-reducing conditions (data not shown).

**Figure 1** Reverse phase high pressure liquid chromatograms of UGP from **a**, urine from a normal adult male **b**, urine pool from a normal pregnancy **c**, urine from a patient with hydatidiform mole, post evacuation and **d**, urine from a patient with ovarian carcinoma. In each case at least three distinct fractions are present.

obtained for fraction 2, the amino acid sequence of samples taken over this peak showed the same N-terminal amino acid sequences. The sequence analysis showing molar yields after each cycle is shown in Table II.

The association of peptide 3 with 2 was also confirmed using immunoassay as well as amino acid sequencing, in the pregnancy material that bound to Con A (contains most of the beta-core fragment activity), the unbound fraction from the DEAE chromatography (contains most of the beta-core fragment reactivity) and finally after gel-filtration of antibody purified material. Fractions obtained by gradient elution of DEAE had only baseline levels of peptide 2 and 3 by immunoassay. The unbound fraction from Con A also contained peptides 2 and 3 (as demonstrated by immunoassay and sequencing) which also were still associated after antibody purification (using immobilised βCF specific antibodies), followed by gel-filtration. However, this product contained at least 10 fold more peptide 3 than peptide 2, which would suggest more than non-specific binding of peptide 3 to the antibody column, especially as we have shown that peptides

**Table I** N-terminal amino acid sequences of the constituents of UGP. The peptide 2 sequence is homologous with that of beta-core fragment and corresponds to residues 6–40 (2a) disulphide linked with residues 55–92 (2b) of hCG-beta subunit. Peptide 1 shows no significant sequence homology with known proteins. Peptide 3 has the N-terminal sequence of non-secretory ribonuclease and eosinophil derived neurotoxin

Peptide 1
1 10
Asp Val Lys (-) Asp Met Glu Val Ser Ser Pro Asp
14
Gly Tyr Thr Ser (-) Arg Leu . . .
Peptide 2
6 10
(a) Arg Pro Arg (-) Arg Pro Ile Asp Ala Thr Leu Ala
55 60
(b) Val Val (-) Asn Tyr Arg Asp Val Arg Phe Glu Ser
20 28
(a) Val Glu Lys Glu Gly (-) Pro Val (-) Ile Thr . . .
70
(b) Ile Arg Leu Pro Gly (-) Pro Arg Gly Val Asn Pro
80
Val Val Ser Tyr . . .
Peptide 3
1 10
Lys Pro Pro Gln Phe Thr (-) Ala Gln (-) Phe Glu
15
Thr Gln (-) Ile (-) Met

(-) No amino acid was detected above background: these positions correspond with cysteine residues of hCG-β subunit, as determined by Bahl.

1 and 3 do not bind βCF antibodies. The association of peptide 1 was more difficult to follow as this appears to be present at lower levels making sequencing more difficult and a specific immunoassay was not available.

The sequence of peptides 1, 2 and 3 were confirmed using purified material from pregnant females and from a patient with a hydatidiform mole. The sequence of peptides 1 and 3 were also confirmed using purified material from a normal male and peptide 1 from a patient with ovarian carcinoma. The presence of peptide 2 in the UGP from the normal male and the ovarian carcinoma patient was confirmed by beta-core fragment immunoassay. The presence of peptides 1, 2 and 3 in material extracted by kaolin adsorption instead of acetone precipitation, was also confirmed using amino acid sequencing. The immunoassays and sequence analyses were tools used to identify the structures present after different purification steps and by themselves do not confirm association. Association is inferred by the continuous presence of the peptides after different purification steps.

### *Immunoassays*

UGP and peptide 2 showed immunological activity in both the RIA and IRMA systems, whilst purified peptides 1 and 3 only showed immunological activity in the RIA system (Figure 2), the reactions of 1 and 3 being linear with concentration and parallel to the UGP response. In the IRMA system, positive response by the peptides 1 and 3 is numerically small and non-parallel and can be discounted in measuring peptide 2. The RIA generated for peptide 3 had a working range of 400–8 ng $ml^{-1}$, 10% cross-reactivity with UGP, 0% cross-reactivity with hCG and β-subunit at 1 μg $ml^{-1}$ and 0% cross-reactivity with beta-core fragment at 2 μg $ml^{-1}$.

### In vitro *bioassay*

UGP and dissociated UGP peptides were tested for bioactivity in the range 1–1000 ng $ml^{-1}$. HCG in the range 3–400 pg $ml^{-1}$ was the control. Some bioactivity was seen at the higher concentrations of UGP which can be attributed to contamination by intact hCG (0.2% by immunoassay). Until this potential contamination can be eliminated comparative

**Table II** Amino acid sequence analysis of immunopurified UGP

| *PEAK and urine source* | *Loading µg* | *Amino acid sequence[a] and yields[b] at each cycle (pmoles)* | | | | | | | | | | | | | | | | | | | | | | | | | | | |
|---|---|---|---|---|---|---|---|---|---|---|---|---|---|---|---|---|---|---|---|---|---|---|---|---|---|---|---|---|---|
| *PEAK 1* | | D | V | K | – | D | M | E | V | S | S | P | D | G | Y | T | | | | | | | | | | | | | |
| Normal male | 2 | 114 | 73 | 37 | | 66 | 36 | 38 | 23 | 41 | 33 | 10 | 21 | 21 | 12 | 5 | | | | | | | | | | | | | |
| Pregnancy | 2 | 133 | 73 | 79 | | 83 | 50 | 60 | 34 | 50 | 33 | 32 | 40 | 31 | 23 | 8 | | | | | | | | | | | | | |
| Hydatidiform mole | 1.5 | 73 | 46 | 13 | | 60 | 29 | 41 | 23 | 15 | 15 | 13 | 40 | 18 | 12 | 7 | | | | | | | | | | | | | |
| Ovarian carcinoma | 1.5 | 62 | 43 | 30 | | 46 | 21 | 29 | 15 | | | | | | | | | | | | | | | | | | | | |
| *PEAK 2A[a]* | | R | P | R | (C) | R | P | I | (N)[c] | A | T | L | A | V | E | K | E | G | (C) | P | V | (C) | I | T | | | | | |
| Pregnancy | 2 | 60 | 71 | 16 | | 40 | 78 | 56 | 8[c] | 63 | 50 | 45 | 46 | 47 | 51 | 39 | 34 | 66[d] | | 34[d] | 21 | | 9 | 10 | | | | | |
| Hydatidiform mole | 2 | 20 | 80 | 20 | | 20 | 43 | 50 | | 44 | 37 | 35 | 37 | 18 | 16 | 12 | 22 | 41[d] | | 24[d] | 9 | | 8 | 7 | | | | | |
| *PEAK 2B[a]* | | V | V | (C) | N | Y | R | D | V | R | F | E | S | I | R | L | P | G | G | P | R | G | V | N | P | V | V | S | Y |
| Pregnancy | 2 | 88 | 113 | | 45 | 105 | 60 | 101 | 75 | 80 | 51 | 74 | 41 | 31 | 40 | 39 | 39 | 66[d] | 52 | 34[d] | 30 | 41 | 10 | 12 | | | | | |
| Hydatidiform mole | 2 | 126 | 99 | | 51 | 53 | 20 | 36 | 41 | 40 | 46 | 53 | 29 | 16 | 30 | 21 | 21 | 41[d] | 32 | 24[d] | 15 | 14 | 10 | 11 | 12 | 11 | 15 | 8 | 11 |
| *PEAK 3[a]* | | K | P | P | Q | F | T | – | A | Q | (W) | F | E | T | Q | (H) | I | (N)[c] | M | | | | | | | | | | |
| Normal male | 2 | 97 | 100 | 78 | 89 | 81 | 53 | | 81 | 46 | | 34 | 36 | 30 | 28 | | 16 | | 10 | | | | | | | | | | |
| Pregnancy | 2 | 57 | 30 | 28 | 34 | 20 | 20 | | 15 | 15 | | 9 | 10 | 7 | 8 | | 4 | | 2 | | | | | | | | | | |
| Hydatidiform mole | 1 | 3 | 27 | 26 | 15 | 15 | 3 | | | | | | | | | | | | | | | | | | | | | | |

[a]Indicates that no amino acid has been determined for this position. An amino acid in brackets indicates that we have not confirmed identity with a published sequence. [b]The yield of amino acid which has increased at each cycle; no correction has been made for a lag. Where no values are given no amino acid was detected above background, allowing for lag in each cycle. [c]Published sequence reports asparagine linked saccharide. The presence of a low yield of aspartate in PEAK 2A is consistent with this. [d]These values represent yields for both chains of PEAK 2.

**Figure 2** **a**, UGP inhibition studies using the AK12 antibody with $^{125}$I-UGP (RIA system). **b**, UGP binding studies using 2C2/$^{125}$I-AK12 (IRMA system). O——O Intact UGP; ●——● UGP-peptide 1; ▲——▲ UGP-peptide 2; ×——× UGP-peptide 3. The RIA system shows inhibition by UGP and all three peptides whilst the IRMA system shows binding with UGP and core-fragment.

studies of biological activity between the peptides will be compromised.

### *Antibody binding data*

The monoclonal antibodies 2C2 and 6D3 only bind UGP or peptide 2 of UGP. This is also the case for antibodies W14 and DR-Pool although they show additional binding to hCG and its beta-subunit. The only antibody preparation which showed any binding with peptides 1 and 3 as well as peptide 2 was the AK12 polyclonal (rabbit) antiserum. Further details on epitope specificities have been published earlier (Kardana *et al.*, 1988, Kardana, 1990; Searle *et al.*, 1984).

### *Isoelectric focusing*

Purified peptide 3 focused characteristically as two bands at pI = 3.5 and pI = 3.8. Peptide 2 migrated towards pI = 9.0 if the gel was visualised before focusing was complete. When focusing had reached completion the peptide 2 band was often lost off the top of the gel, indicating a pI greater than the range of the gel. Insufficient purified peptide 1 was available to visualise on gels.

### *Urine chromatography*

Fractions from the gel-filtration of normal male urine when analysed for peptide 3 by immunoassay, demonstrated only one region of immunoreactivity. This was at Mr = 15,000, the same as that for UGP. There was no additional peak of immunoreactivity for peptide 3 in the lower molecular weight region.

### Discussion

HPLC separation of purified preparations of UGP from different source material showed at least three distinct peaks in each case. The amino acid sequence of peptide 1 did not identify it as a previously known sequence. Peptide 2 was identified as the beta core-fragment of hCG, and peptide 3 appears to be related to non-secretory ribonuclease U (RN-ase U) but the final structure remains to be elucidated.

Initially, reverse phase HPLC was used as a final 'clean-up' of the beta-core fragment preparations and the additional peptides (1 and 3) were regarded as contaminants. However, successive UGP purifications, from the urine of patients with different malignancies, also showed the presence of the same two peptides. It was felt therefore, this warranted further investigation.

The association of peptide 3 with beta core-fragment, in UGP purified from pregnancy material, was confirmed by immunoassay and sequencing, at each step, after gel-filtration followed by Con-A purification followed by ion-exchange chromatography followed by purification using immobilised antibodies and finally gel-filtration (Sephadex G-100) again. The association of peptide 1 with 2 and 3 was confirmed by sequencing at the final step, after gel-filtration, antibody affinity purification and gel-filtration using Sephadex G-25. Dissociation of peptides 1, 2 and 3 was obtained under the denaturing conditions of reverse phase HPLC.

In order to eliminate the possibility that the association of these peptides occurred as a result of the initial acetone precipitation step in the extraction procedure (Kardana *et al.*, 1988), an alternative concentration step was used which relied on adsorption to kaolin. However, although the UGP recovery was not as good using the kaolin method (only about 25% of the acetone precipitation recovery), all three peptides were again detected. These data are strong indicators of association, although they do not conclusively eliminate the possibility of co-purification.

When normal male urine was chromatographed and analysed for peptide 3 by immunoassay, only one peak of immuno-reactivity was obtained, which was in the region of UGP (Mr = 15,000). If the association of beta-core fragment with peptide 3 occurred at the kidney level, some unassociated peptide 3 might be expected, as the beta-core fragment content of normal urine is low (Nam *et al.*, 1990).

The cervical carcinoma cell lines DOT and Caski were reported as secreting a molecule with beta immunoreactivity, that was larger than beta subunit, had the C-terminal peptide missing and did not react with antibodies raised to the alpha subunit of hCG (Hussa *et al.*, 1986). It has been shown that the major form of beta-core fragment in pregnancy serum exists as a large molecular weight complex (Mr >60,000) (Kardana & Cole, 1990). This complex, which does not appear to be the result of non-specific binding of beta-core fragment to serum proteins, can be dissociated with 3 M ammonium thiocyanate. The identity of the associated macromolecules is as yet unknown, but they are not hCG related. UGP could possibly be the large molecules secreted by the Dot and Caski cell lines, or the degradatory product of serum beta-core fragment complex after processing by either the liver or kidneys, and variable extent of degradation could lead to variable stoichiometry.

Isoelectric focusing of peptide 2 (BCF) gives it a pI >9.0. The high pI value observed would be consistent with a $\beta$-core fragment lacking sialic acid, of the type fully investigated by previous workers (Blithe *et al.*, 1989) in that within hCG and its subunits desialylated molecules have a higher pI than their fully sialylated counterparts. It has been shown that the glomerular basement membrane (GBM) of the kidney has a large distribution of anionic sites (Brenner *et al.*, 1977; Caulfield & Farquhar, 1976; Chang *et al.*, 1975). These anionic sites are due to sialo-glycoproteins present within the GBM which repel anions, thus hindering their passage through the GBM into the urinary filtrate and keeping them in the plasma circulation. It has also been shown that strong cations with pI $>8.8$ are rapidly cleared from the circulation and have been demonstrated within the basement membrane, but they cannot be detected in the urinary spaces (Rennke *et al.*, 1975; Venkatachalam & Rennke, 1978). These are thought to be taken up and processed in lysosomes. Therefore beta-core fragment with a pI $>9.0$ would also not be expected to be found in the urinary filtrate, except maybe at low levels. It has been reported (Lefort *et al.*, 1986) that hCG and hCG beta-subunit, when infused into rats appears as intact hCG or beta-subunit in serum, which is catabolised and appears as a fragment that has anti-beta immunoreactivity but no anti-CTP immunoreactivity in liver and kidney homogenates, whilst in the urine a small molecular form which has only immunoreactivity with the CTP antibodies was detected. In addition, the beta immunoreactive form that was detected in the kidney homogenates, appeared after 30 min of infusion with hCG, but the amount of this fragment in the kidney, remained fairly constant even after 6 h. Virtually no beta-immunoreactive fragment was detected in the rat urine. This data supports the above statement that if beta-core fragment was circulating unassociated, it would be removed and retained by the kidney GBM and would not filter into the urine.

However beta-core fragment is detected at high levels in pregnancy and trophoblastic urine (Blithe *et al.*, 1988; Kato & Braunstein, 1988; Papapetrou & Nicopoulou, 1986). Similarly, peptide 3 with its strong anionic charge should be repelled from the GBM and thus the urinary filtrate. Perhaps the association of peptides 1, 2 and 3 as UGP, modifies the overall charge of the molecule and thus enables UGP to pass through the GBM into the urine.

Further work needs to be done to determine where UGP association occurs and if there is any link with the serum beta-core fragment complex or the larger molecules detected in cervical carcinoma culture medium. Perhaps the additional peptides in UGP could be used as a starting point to try and identify the associated macromolecules in the serum beta-core fragment complex.

In conclusion, the association of peptides 1, 2 and 3 as demonstrated by the techniques we have used, appear to be specific. However, whether the association is specific or a co-elution problem, this report should alert other investigators purifying beta-core fragment, using these standard biochemical methodologies.

The two immunoassays (RIA and IRMA) used to monitor UGP have different characteristics. The AK12 RIA has antibodies to peptides 1, 2 and 3 so when all three peptides are iodinated (as UGP) this system can measure UGP and any free peptides, if they occur. However when only peptide three was iodinated, a specific assay for peptide 3 resulted that did not cross-react with peptides 1 and 2.

The 2C2 IRMA used a capture antibody specific for beta-core fragment (peptide 2) and radioiodinated AK12 as tracer. These measurements could be regarded as values of UGP, or amplified beta-core fragment, as additional tracer could bind to peptides 1 and 3 when they are associated with beta-core fragment as UGP.

An analysis of the results obtained on urine samples from patients with trophoblastic and non-trophoblastic neoplasms, using these immunoassays, will be used to assess the value of UGP, as opposed to beta-core fragment, as a tumour marker.

### References

AKAR, A.H., WEHMANN, R.E., BLITHE, D.L. BLACKER, C. & NISULA, B.C. (1988). A radioimmunoassay for the core fragment of the human chorionic gonadotrophin β-subunit. *J. Clin. Endocrinol. Metab.*, **66**, 538–545.

ALBERT, A. (1956). Human urinary gonadotropin. In *Recent Progress in Hormone Res.*, **12**, 227–296.

BAHL, O.P., CARLSEN, R.B., BELLISARIO, R. & SWAMINATHAN, N. (1972). Human chorionic gonadotrophin: amino acid sequence of the α- and β-subunits. *Biochem. Biophys. Res. Commun.*, **48**, 416–422.

BEINTEMA, J.J., HOFSTEENGE, J., IWAMA, M., MORITA, T., OHGI, K., IME, M., SUGIYAMA, R.H., SCHIEVEN, G.L., DEKKER, C.A. & GLITZ, D.G. (1988). Amino acid sequence of the nonsecretory ribonuclease of human urine. *Biochemistry*, **27**, 4530–4538.

BIRKEN, S., ARMSTRONG, E.G., KOLKS, M.A.G., COLE, L.A., AGOSTO, G.M., KRICHEVSKY, A., VAITUKAITIS, J.L. & CANFIELD, R.E. (1988). Structure of the human chorionic gonadotrophin β-subunit fragment from pregnancy urine. *Endocrinology*, **123**, 572–783.

BLITHE, D.L., AKAR, A.H., WEHMANN, R.E. & NISULA, B.C. (1988). Purification of β-core fragment from pregnancy urine and demonstration that its carbohydrate moieties differ from those of native human chorionic gonadotrophin-β. *Endocrinology*, **122**, 173–180.

BLITHE, D.L., WEHMANN, R.E. & NISULA, B.C. (1989). Carbohydrate composition of β-core. *Ibid.*, **125**, 2267–2272.

BRENNER, B.M., BOHRER, M.P., BAYLIS, C. & DEEN, W.M. (1977). Determinants of glomerular permselectivity: Insights derived from observations *in vivo*. *Kidney Int.*, **12**, 229–237.

CAULFIELD, J.P. & FARQUHAR, M.G. (1976). Distribution of anionic sites in glomerular basement membranes: their possible role in filtration and attachment. *Proc. Natl. Acad. Sci. USA*, **73**, 1646–1650.

CHANG, R.L.S., DEEN, W.M., ROBERTSON, C.R. & BRENNER, B.M. (1975). Permselectivity of the glomerular capillary wall: III. Restricted transport of polyanions. *Kidney Int.*, **8**, 212–218.

COLE, L.A., WANG, Y., ELLIOTT, M., LATIF, M., CHAMBERS, J.T. & SCHWARTZ, P.E. (1988). Urinary human chorionic gonadotrophin free beta-subunit and beta-core fragment: a new marker of gynaecological cancers. *Cancer Res.*, **48**, 1356–1360.

FRAKER, P.J. & SPECK, J.C.Jr. (1978). Protein and cell membrane iodinations with a sparingly soluble chloroamide, 1, 3, 4, 6-tetrachloro-diphenylglycoluril. *Biochem. Biophys, Res. Commun.*, **80**, 849–857.

HUSSA, R.O., FEIN, H.G., PATTILLO, R.A., NAGELBERG, S.B., ROSEN, S.W., WEINTRAUB, B.D., PERINI, F., RUDDON, R.W. & COLE, L.A. (1986). A distinctive form of human chorionic gonadotrophin β-subunit-like material produced by cervical carcinoma cells. *Cancer Res.*, **46**, 1948–1954.

KARDANA, A. (1990). *Extraction, purification, characterisation and clinical evaluation of urinary gonadotrophin peptide (UGP) as a tumour marker*. PhD. London University 100.

KARDANA, A., TAYLOR, M.E., SOUTHALL, P.J., BOXER, G.M., ROWAN, A.J. & BAGSHAWE, K.D. (1988). Urinary gonadotrophin peptide – isolation and purification, and its immunohistochemical distribution in normal and neoplastic tissues. *Br. J. Cancer*, **58**, 281–286.

KARDANA, A., TAYLOR, M.E., ROWAN, A.J., READ, D.A. & BAGSHAWE, K.D. (1989). Characterisation of antibodies to urinary gonadotrophin peptide. *J. Immunol. Methods*,. **118**, 53–58.

KARDANA, A. & COLE, L.A. (1990). Serum hCG β-core fragment is masked by associated macromolecules. *J. Clin. Endocrinol. Metab.*, **71**, 1393–1395.

KATO, Y. & BRAUNSTEIN, G.D. (1988). β-Core fragment is a major form of immunoreactive urinary chorionic gonadotrophin in human pregnancy. *J. Clin. Endocrinol. Metab.*, **66**, 1197–1201.

LEFORT, G.P., STOLK, J.M. & NISULA, B.C. (1986). Renal metabolism of the β-subunit of human choriogonadotropin in the rat. *Endocrinology*, **119**, 924–931.

NAM, J.H., COLE, L.A., CHAMBERS, J.T. & SCHWARTZ, P.E. (1990). Urinary gonadotrophin fragment, a new tumour marker: I. Assay development and cancer specificity. *Gynecol. Oncol.*, **36**, 383–390.

PAPAPETROU, P.D. & NICOPOULOU, S.C. (1986). The origin of a human chorionic gonadotrophin β-subunit-core fragment excreted in the urine of patients with cancer. *Acta Endocrinol.*, **112**, 415–422.

RENNKE, H.G., COTRAN, R.S. & VENKATACHALAM, M.A. (1975). Role of molecular charge in glomerular permeability. *J. Cell Biol.*, **67**, 638–646.

SEARLE, F., PARTRIDGE, C.S., KARDANA, A., GREEN, A.J., BUCKLEY, R.J., BEGENT, R.H.J. & RAWLINS, G.A. (1984). Preparation and properties of a mouse monoclonal antibody (W14A) to human chorionic gonadotrophin. *Int. J. Cancer*, **33**, 429–434.

VAN DAMME, M.P., ROBERTSON, D.M. & DICZFALUSY, E. (1974). An improved *in vitro* bioassay method for measuring luteinising hormone (LH) activity using mouse Leydig cell preparations. *Acta Endocrinol.*, **77**, 655–671.

VENKATACHALAM, M.A. & RENNKE, H.G. (1978). The structural and molecular basis of glomerular filtration. *Circ. Res.*, **43**, 337–347.

WEHMANN, R.E. & NISULA, B.C. (1980). Characterisation of a discrete degradation product of the human chorionic gonadotrophin beta-subunit in humans. *J. Clin. Endocrinol. Metab.*, **51**, 101–105.